# Supplementary material for: Plants capable of selfing are more likely to become naturalized
Source: Nat Commun. 2016 Oct 31;7:13313. doi: 10.1038/ncomms13313 (PMC5095580; doi:10.1038/ncomms13313)
Supplement: Supplementary Information — Supplementary Figures 1-5 and Supplementary Tables 1-4 [file ncomms13313-s1.pdf]

Supplementary Figures

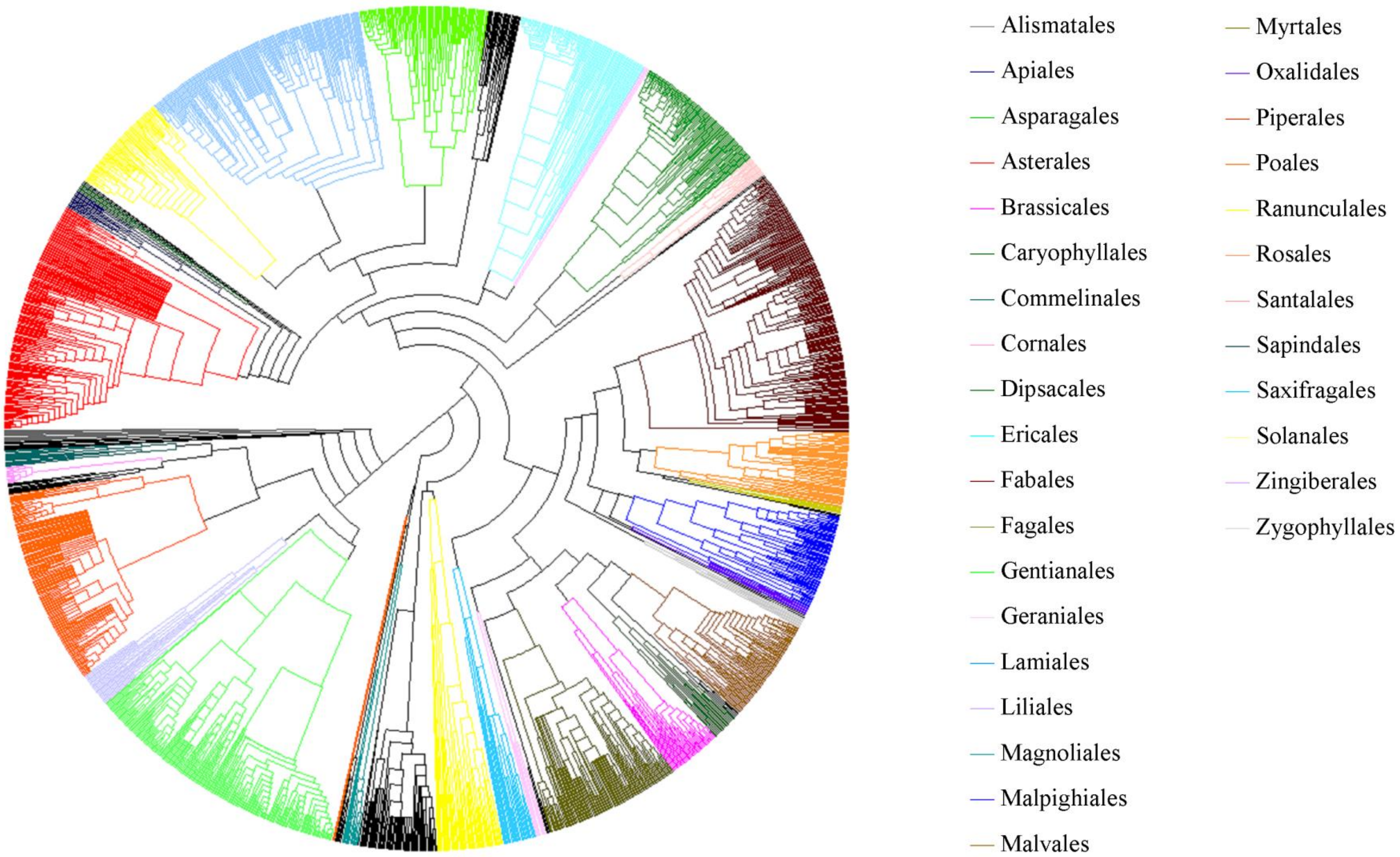

Supplementary Figure 1 A phylogenetic tree of the 1,752 angiosperm species included in our global database on breeding systems. Different colors represent orders according to the Angiosperm Phylogeny Group III system.

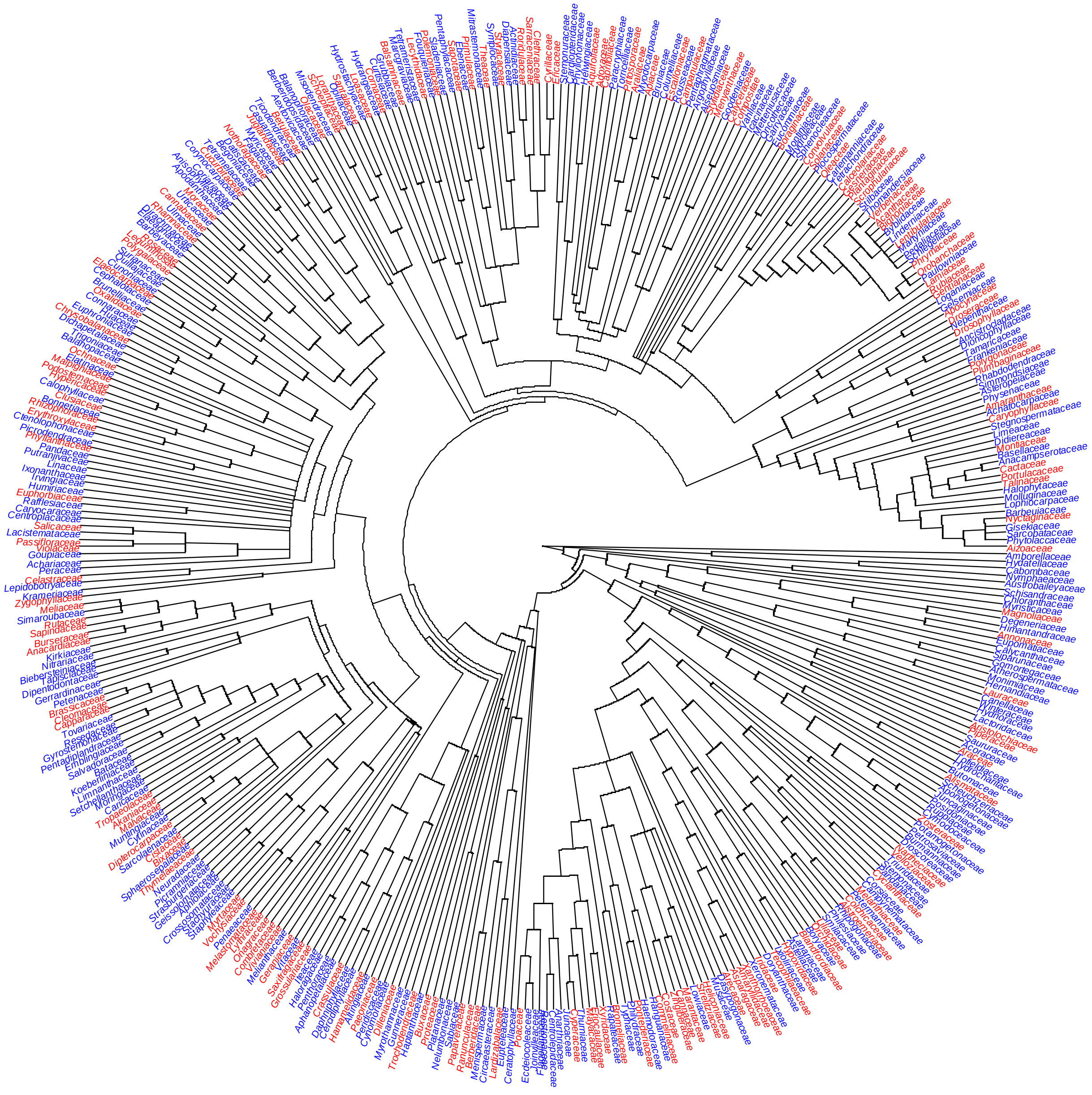

Supplementary Figure 2 A phylogenetic tree of all angiosperm families. Families represented in our global database on breeding systems are depicted in red; families not represented in the database are depicted in blue.

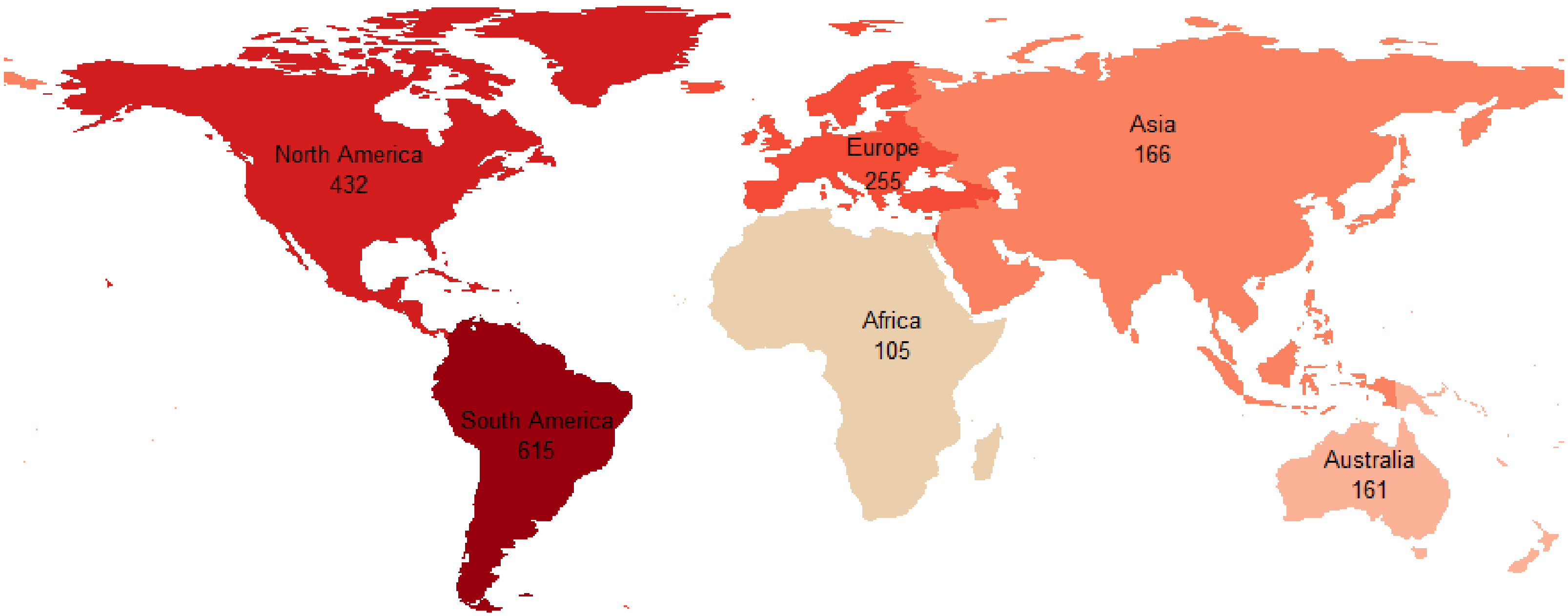

Supplementary Figure 3 Number of species from each continent, for which breeding system data was compiled in our global database on breeding systems. Light red indicates the smallest number of species (i.e. Africa) and dark red indicates the largest number of species (i.e. South America).

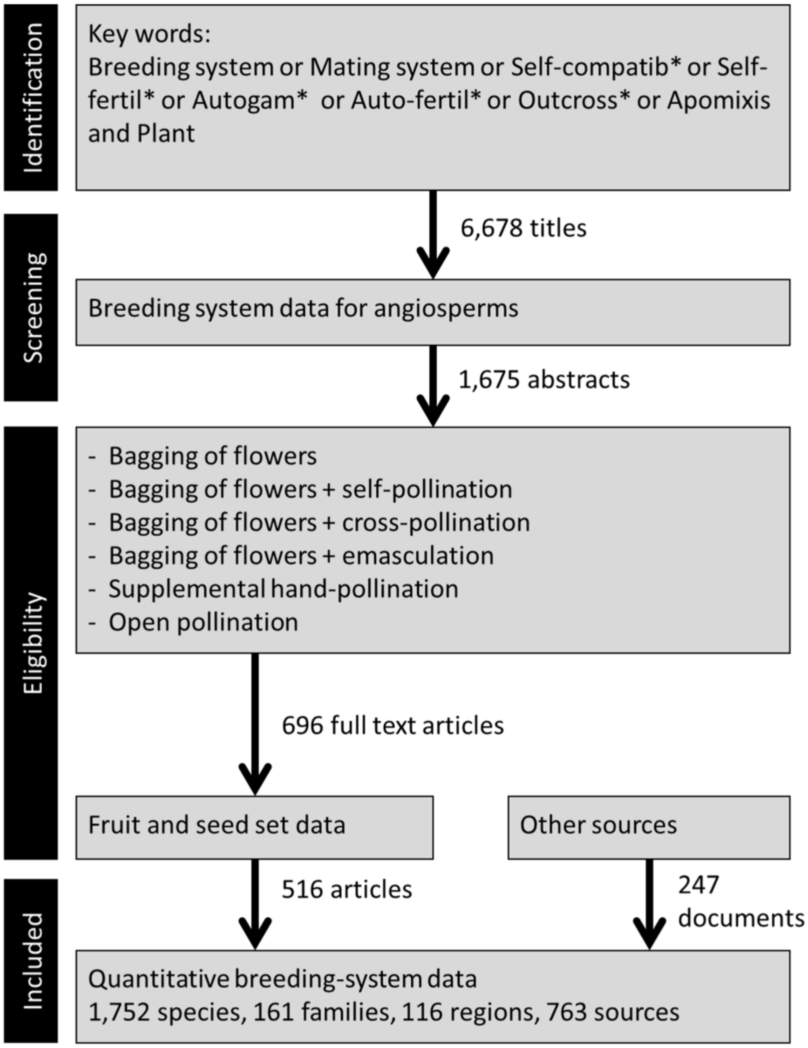

Supplementary Figure 4 Procedure used for selection of studies for the compilation of our global database on breeding systems using Web of Science and other sources.

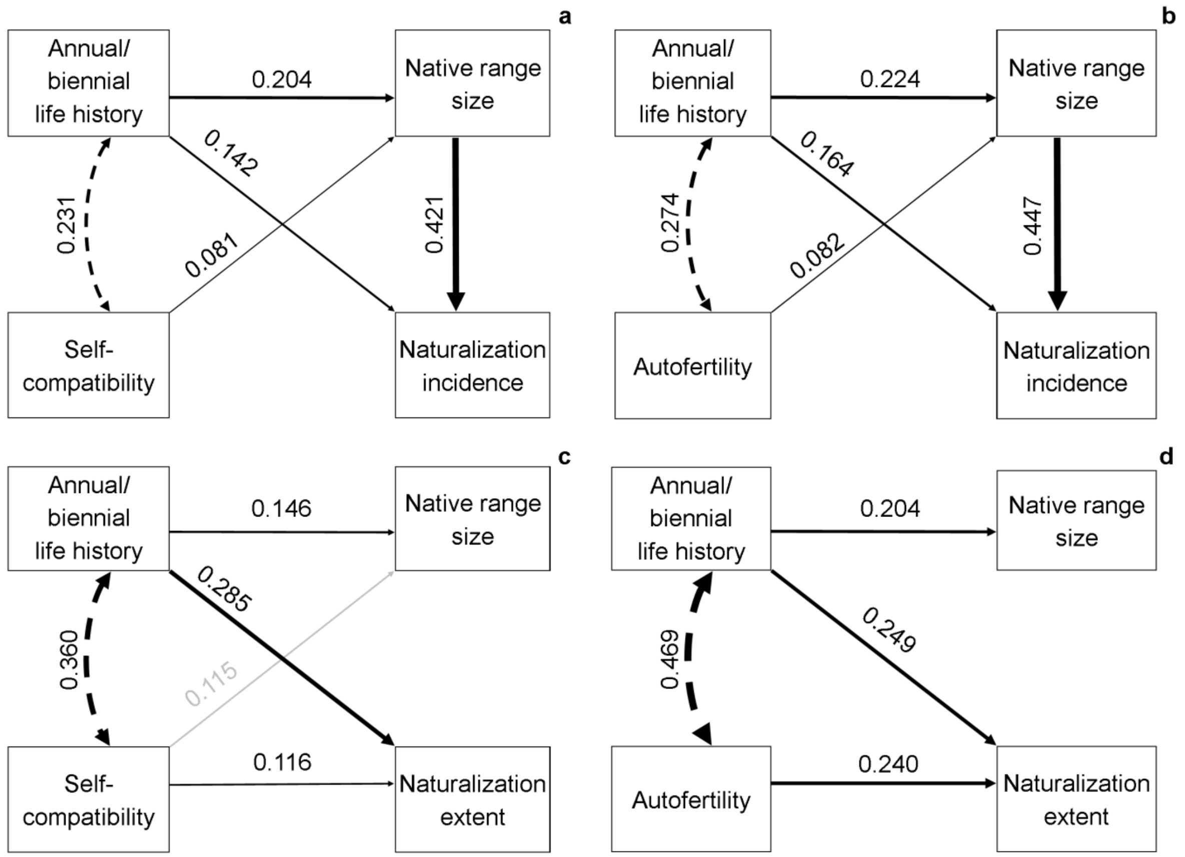

Supplementary Figure 5 Direct and indirect effects of selfing ability on the global naturalization of alien plant species. Path analyses with one average selfing ability index per species. **a**, Effects of self-compatibility on naturalization incidence (expressed as being naturalized somewhere or not); *n* = 1115, no. parameters = 7, Comparative Fit Index (CFI) = 0.989, Root Mean Square Error of Approximation (RMSEA) = 0.064, $R^2_{\text{naturalization incidence}}$=0.22. **b**, Effects of autofertility on naturalization incidence; *n* = 828, no. parameters = 7, CFI = 0.983, RMSEA = 0.079, $R^2_{\text{naturalization incidence}}$=0.27. **c**, Effects of self-compatibility on the extent of naturalization (number of regions where the species is naturalized); *n* = 274, no. parameters = 8, CFI = 1.000, RMSEA = 0.000, $R^2_{\text{naturalization extent}}$=0.13. **d**, Effects of autofertility on the extent of naturalization; *n* = 195, no. parameters = 8, CFI = 1.000, RMSEA = 0.000, $R^2_{\text{naturalization extent}}$=0.18. Indices were calculated using fruit set from our global breeding-system database. Sample sizes refer to the number of species. Curved dashed lines with two-headed arrows indicate bivariate correlations between variables; solid lines with one-headed arrow indicate directed paths connecting the variables. The thickness of each path is proportional to the value of the path coefficient. Only path coefficients that are significant (z-test, $p < 0.05$; black) and marginally significant ($p < 0.1$; grey) are shown.

Supplementary Tables

Supplementary Table 1 Results of four non-phylogenetic logistic and linear regressions testing the global naturalization of alien species in relation to selfing ability (measured as self-compatibility and autofertility indices), native range size, annual/biennial vs. perennial life history, and the interaction between life history and selfing ability. Global naturalization was measured as naturalization incidence outside the native range (expressed as being naturalized somewhere or not) and naturalization extent (natural log-transformed number of regions where the species is naturalized). Native range size was measured as the number of TDWG level-2 regions. Self-compatibility and autofertility indices were calculated using fruit set from our global breeding-system database. Sample sizes refer to the total number of species from individual studies in the breeding-system database. [a]Logistic regressions. [b]Linear models. [c]Variables were rescaled to have a mean of zero and a standard deviation of one. [d]Total explained variance by the variables in the model, and the relative importance of each variable calculated as the difference in deviance or sum of squares of residuals between the full model and a model without the variable of interest.

Response variables	Naturalization incidence[a]					Naturalization extent[b]				
Explanatory variables	Estimates	SE	z	p	R^{2d}	Estimates	SE	t	p	R^{2d}
Analysis with self-compatibility	*n* = 1181				0.21	*n* = 295				0.12
Intercept	-1.222	0.077	-15.72	<0.0001	-	2.164	0.107	20.15	<0.0001	-
Native range size (no. regions) [c]	0.850	0.085	10.04	<0.0001	85.62	0.070	0.096	0.73	0. 467	1.85
Annual/biennial[c]	0.702	0.461	1.52	0.128	11.18	1.661	0.474	3.50	<0.001	77.15
Self-compatibility index[c]	-0.087	0.077	-1.14	0.256	2.15	0.182	0.107	1.70	0.090	18.53
Annual/biennial[c] x Self-compatibility[c]	0.515	0.414	1.24	0.213	1.04	-0.377	0.448	-0.84	0.400	2.47
Analysis with autofertility	*n* = 866				0.25	*n* = 203				0.16
Intercept	-1.345	0.092	-14.55	<0.0001	-	2.348	0.128	18.35	<0.0001	-
Native range size (no. regions)[c]	0.885	0.101	8.71	<0.0001	77.59	-0.017	0.119	-0.14	0.885	0.09
Annual/biennial[c]	0.864	0.369	2.34	0.019	16.13	1.178	0.383	3.07	0.002	50.32
Autofertility[c]	0.032	0.093	0. 35	0.729	3.35	0.443	0.131	3.37	<0.001	49.25
Annual/biennial[c] x Autofertility[c]	0.501	0.262	1.91	0.056	2.93	-0.083	0.300	-0.28	0.782	0.33

Supplementary Table 2 Number of plant taxa of which breeding-system data were documented

	Pollinator exclusion	Self-pollination	Cross-pollination	Open pollination	Apomixis	Pollen supplementation
Percent fruit set	1165	1293	1230	964	403	147
Seeds per flower	654	731	486	443	378	90
(% calculated)	(33.38 %)	(56.63 %)	(53.90 %)	(60.95 %)	(38.36 %)	(37.78 %)

Supplementary Table 3 References used to add the remaining 486 species to a tree with 1266 tips in order to construct a phylogenetic tree of the 1752 species in the breeding system database

Taxa	References
Acanthaceae	McDade, L.A. & Moody, M.L. Phylogenetic relationships among Acanthaceae: evidence from noncoding trnL-trnF chloroplast DNA sequences. *Am. J. Bot.* **86**, 70-80 (1999).
	McDade, L.A., Masta, S.E., Moody, M.L. & Waters, E. Phylogenetic relationships among Acanthaceae: evidence from two genomes. *Syst. Bot.* **25**, 106-121 (2000).
Amaryllidaceae	Ito, M., Kawamoto, A., Kita, Y., Yukawa, T. & Kurita, S. Phylogenetic relationships of Amaryllidaceae based on matK sequence data. *J. Plant Res.* **112**, 207-216 (1999).
Annonaceae	Chatrou, L.W., Pirie, M.D., Erkens, R.H., Couvreur, T.L., Neubig, K.M., Abbott, J.R., Mols, J.B., Maas, J.W., Saunders, R.M. & Chase, M.W. A new subfamilial and tribal classification of the pantropical flowering plant family Annonaceae informed by molecular phylogenetics. *Bot. J. Linn. Soc.* **169**, 5-40 (2012).
Apiaceae	Downie, S.R., Katz-Downie, D.S. & Watson, M.F. A phylogeny of the flowering plant family Apiaceae based on chloroplast DNA rpl16 and rpoC1 intron sequences: towards a suprageneric classification of subfamily Apioideae. *Am. J. Bot.* **87**, 273-292 (2000).
Apocynaceae	Sennblad, B. & Bremer, B. Classification of Apocynaceae s. 1. according to a new approach combining Linnaean and Phylogenetic taxonomy. *Syst. Biol.* **51**, 389-409 (2002).
Araceae	Angiosperm Phylogeny Website version 13 (http://www.mobot.org/MOBOT/research/APweb/)
Asparagaceae	Kim, D.-K., Kim, J.S. & Kim, J.-H. The phylogenetic relationships of Asparagales in Korea based on five plastid DNA regions. *J. Plant Biol.* **55**, 325-341 (2012).
Asteraceae	Panero, J.L. & Funk, V.A. Toward a phylogenetic subfamilial classification for the Compositae (Asteraceae). *P. Biol. Soc. Wash.* **115**, 909-922 (2002).
	Panero, J.L. & Funk, V.A. The value of sampling anomalous taxa in phylogenetic studies: major clades of the Asteraceae revealed. *Mol. Phylogenet. Evol.* **47**, 757-782 (2008).
	Goertzen, L.R., Cannone, J.J., Gutell, R.R. & Jansen, R.K., ITS secondary structure derived from comparative analysis: implications for sequence alignment and phylogeny of the Asteraceae. *Mol. Phylogenet. Evol.* **29**, 216-234 (2003).
Bignoniaceae	Olmstead, R.G., Zjhra, M.L., Lohmann, L.G., Grose, S.O. & Eckert, A.J. A molecular phylogeny and classification of Bignoniaceae. *Am. J. Bot.* **96**, 1731-1743 (2009).
Blandfordia	Angiosperm Phylogeny Website version 13 (http://www.mobot.org/MOBOT/research/APweb/)
Boraginaceae	Långström, E. & Chase, M. Tribes of Boraginoideae (Boraginaceae) and placement of Antiphytum, Echiochilon, Ogastemma

Taxa	References
	and Sericostoma: a phylogenetic analysis based on atpB plastid DNA sequence data. *Plant Syst. Evol.* 234, 137-153 (2002).
Brassicaceae	Bailey, C.D., Koch, M.A., Mayer, M., Mummenhoff, K., O'Kane, S.L., Warwick, S.I., Windham, M.D. & Al-Shehbaz, I.A. Toward a global phylogeny of the Brassicaceae. *Mol. Biol. Evol.* **23**, 2142-2160 (2006).
Bromeliaceae	Barfuss, M.H., Samuel, R., Till, W. & Stuessy, T.F. Phylogenetic relationships in subfamily Tillandsioideae (Bromeliaceae) based on DNA sequence data from seven plastid regions. *Am. J. Bot.* **92**, 337-351 (2005).
	Terry, R., Brown, G. & Olmstead, R. Examination of subfamilial phylogeny in Bromeliaceae using comparative sequencing of the plastid locus ndhF. *Am. J. Bot.* **84**, 664-664 (1997).
Cactaceae	Butterworth, C.A., Cota-Sanchez, J.H. & Wallace, R.S. Molecular systematics of tribe Cacteae (Cactaceae: Cactoideae): a phylogeny based on rpl16 intron sequence variation. *Syst. Bot.* **27**, 257-270 (2002).
	Nyffeler, R. Phylogenetic relationships in the cactus family (Cactaceae) based on evidence from trnK/matK and trnL-trnF sequences. *Am. J. Bot.* **89**, 312-326 (2002).
Campanulaceae	Antonelli, A. Higher level phylogeny and evolutionary trends in Campanulaceae subfam. Lobelioideae: Molecular signal overshadows morphology. *Mol. Phylogenet. Evol.* **46**, 1-18 (2008).
	Eddie, W., Shulkina, T., Gaskin, J., Haberle, R. & Jansen, R. Phylogeny of Campanulaceae s. str. inferred from ITS sequences of nuclear ribosomal DNA. *Ann. Mo. Bot. Gard.* **90**, 554-575 (2003).
Caryophyllaceae	Fior, S., Karis, P.O., Casazza, G., Minuto, L. & Sala, F. Molecular phylogeny of the Caryophyllaceae (Caryophyllales) inferred from chloroplast matK and nuclear rDNA ITS sequences. *Am. J. Bot.* **93**, 399-411 (2006).
Combretaceae	Angiosperm Phylogeny Website version 13 (http://www.mobot.org/MOBOT/research/APweb/)
Commelinaceae	Evans, T.M., Sytsma, K.J., Faden, R.B. & Givnish, T.J. Phylogenetic relationships in the Commelinaceae: II. A cladistic analysis of rbcL sequences and morphology. *Syst. Bot.* **28**, 270-292 (2003).
Convolvulaceae	Stefanovic, S., Austin, D.F. & Olmstead, R.G. Classification of Convolvulaceae: a phylogenetic approach. *Syst. Bot.* **28**, 791-806 (2003).
Cyperaceae	Simpson, D.A., Muasya, A.M., Alves, M.V., Bruhl, J.J., Dhooge, S., Chase, M.W., Furness, C.A., Ghamkhar, K., Goetghebeur, P. & Hodkinson, T. Phylogeny of Cyperaceae based on DNA sequence data-a new rbcL analysis. *Aliso* **23**, 72-83 (2007).
Dipterocarpaceae	Dayanandan, S., Ashton, P.S., Williams, S.M. & Primack, R.B., Phylogeny of the tropical tree family Dipterocarpaceae based on nucleotide sequences of the chloroplast rbcL gene. *Am. J. Bot.* **86**, 1182-1190 (1999).
	Tsumura, Y., Kawahara, T., Wickneswari, R. & Yoshimura, K., Molecular phylogeny of Dipterocarpaceae in Southeast Asia using RFLP of PCR-amplified chloroplast genes. *Theor. Appl. Genet.* **93**, 22-29 (1996).
Elaeocarpaceae	Angiosperm Phylogeny Website version 13 (http://www.mobot.org/MOBOT/research/APweb/)
Ericaceae	Kron, K.A., Judd, W., Stevens, P., Crayn, D., Anderberg, A., Gadek, P., Quinn, C. & Luteyn, J. Phylogenetic classification of Ericaceae: molecular and morphological evidence. *Bot. Rev.* **68**, 335-423 (2002).

Taxa	References
Fabaceae	Wojciechowski, M.F., Lavin, M. & Sanderson, M.J. A phylogeny of legumes (Leguminosae) based on analysis of the plastid matK gene resolves many well-supported subclades within the family. *Am. J. Bot.* **91**, 1846-1862 (2004).
Gentianaceae	Mansion, G. & Struwe, L. Generic delimitation and phylogenetic relationships within the subtribe Chironiinae (Chironieae: Gentianaceae), with special reference to Centaurium: evidence from nrDNA and cpDNA sequences. *Mol. Phylogenet. Evol.* **32**, 951-977 (2004).
	Yuan, Y.-M. & Küpfer, P. Molecular phylogenetics of the subtribe Gentianinae (Gentianaceae) inferred from the sequences of internal transcribed spacers (ITS) of nuclear ribosomal DNA. *Plant Syst. Evol.* **196**, 207-226 (1995).
Gesneriaceae	Moller, M. & Cronk, Q. Origin and relationships of Saintpaulia (Gesneriaceae) based on ribosomal DNA internal transcribed spacer (ITS) sequences. *Am. J. Bot.* **84**, 956-956 (1997).
	Zimmer, E.A., Roalson, E.H., Skog, L.E., Boggan, J.K. & Idnurm, A. Phylogenetic relationships in the Gesnerioideae (Gesneriaceae) based on nrDNA ITS and cpDNA trnL-F and trnE-T spacer region sequences. *Am. J. Bot.* **89**, 296-311 (2002).
Iridaceae	Reeves, G., Chase, M.W., Goldblatt, P., Rudall, P., Fay, M.F., Cox, A.V., Lejeune, B. & Souza-Chies, T. Molecular systematics of Iridaceae: evidence from four plastid DNA regions. *Am. J. Bot.* **88**, 2074-2087 (2001).
Lamiaceae	Wagstaff, S.J., Hickerson, L., Spangler, R., Reeves, P.A. & Olmstead, R.G. Phylogeny in Labiatae sl, inferred from cpDNA sequences. *Plant Syst. Evol.* **209**, 265-274 (1998).
Lauraceae	Rohwer, J.G. Toward a phylogenetic classification of the Lauraceae: evidence from matK sequences. *Syst. Bot.* **25**, 60-71 (2000).
Loranthaceae	Vidal-Russell, R. & Nickrent, D.L. Evolutionary relationships in the showy mistletoe family (Loranthaceae). *Am. J. Bot.* **95**, 1015-1029 (2008).
Lythraceae	Graham, S.A., Hall, J., Sytsma, K. & Shi, S.h. Phylogenetic analysis of the Lythraceae based on four gene regions and morphology. *Int. J. Plant Sci.* **166**, 995-1017 (2005).
Magnolia	Azuma, H., Thien, L.B. & Kawano, S. Molecular phylogeny of Magnolia (Magnoliaceae) inferred from cpDNA sequences and evolutionary divergence of the floral scents. *J. Plant Res.* **112**, 291-306 (1999).
Malvaceae	Alverson, W.S., Whitlock, B.A., Nyffeler, R., Bayer, C. & Baum, D.A. Phylogeny of the core Malvales: evidence from ndhF sequence data. *Am. J. Bot.* **86**, 1474-1486 (1999).
Melastomataceae	Clausing, G. & Renner, S.S. Molecular phylogenetics of Melastomataceae and Memecylaceae: implications for character evolution. *Am. J. Bot.* **88**, 486-498 (2001).
	Michelangeli, F.A., Guimaraes, P.J., Penneys, D.S., Almeda, F. & Kriebel, R. Phylogenetic relationships and distribution of new world Melastomeae (Melastomataceae). *Bot. J. Linn. Soc.* **171**, 38-60 (2013).
	Renner, S.S. Phylogeny and classification of the Melastomataceae and Memecylaceae. *Nord. J. Bot.* **13**, 519-540 (1993).
Myrtaceae	Wilson, P.G., O'brien, M., Heslewood, M. & Quinn, C. Relationships within Myrtaceae sensu lato based on a matK

Taxa	References
	phylogeny. *Plant Syst. Evol.* **251**, 3-19 (2005).
Onagraceae	Levin, R.A., Wagner, W.L., Hoch, P.C., Nepokroeff, M., Pires, J.C., Zimmer, E.A. & Sytsma, K.J. Family-level relationships of Onagraceae based on chloroplast rbcL and ndhF data. *Am. J. Bot.* **90**, 107-115 (2003).
Orchidaceae	Cameron, K.M., Chase, M.W., Whitten, W.M., Kores, P.J., Jarrell, D.C., Albert, V.A., Yukawa, T., Hills, H.G. & Goldman, D.H. A phylogenetic analysis of the Orchidaceae: evidence from rbcL nucleotide sequences. *Am. J. Bot.* **86**, 208-224 (1999).
Orobanchaceae	Bennett, J.R. & Mathews, S. Phylogeny of the parasitic plant family Orobanchaceae inferred from phytochrome A. *Am. J. Bot.* **93**, 1039-1051 (2006).
Pandanales	Angiosperm Phylogeny Website version 13 (http://www.mobot.org/MOBOT/research/APweb/)
Papaveraceae	Hoot, S., Kadereit, J., Blattner, F., Jork, K., Schwarzbach, A. & Crane, P. Data congruence and phylogeny of the Papaveraceae sl based on four data sets: atpB and rbcL sequences, trnK restriction sites, and morphological characters. *Syst. Bot.* **22**, 575-590 (1997).
	Schwarzbach, A.E. & Kadereit, J.W. Phylogeny of prickly poppies, Argemone (Papaveraceae), and the evolution of morphological and alkaloid characters based on ITS nrDNA sequence variation. *Plant Syst. Evol.* **218**, 257-279 (1999).
Piperales	Angiosperm Phylogeny Website version 13 (http://www.mobot.org/MOBOT/research/APweb/)
Poaceae	Grass Phylogeny Work Group, Barker, N.P., Clark, L.G., Davis, J.I., Duvall, M.R., Guala, G.F., Hsiao, C., Kellogg, E.A., Linder, H.P. & Mason-Gamer, R.J. Phylogeny and subfamilial classification of the grasses (Poaceae). *Ann. Mo. Bot. Gard.* **88**, 373-457 (2001).
Polemoniaceae	Bell, C.D. & Patterson, R.W. Molecular phylogeny and biogeography of Linanthus (Polemoniaceae). *Am. J. Bot.* **87**, 1857-1870 (2000).
Polygalaceae	Abbott, J.R. Phylogeny of the Polygalaceae and a revision of Badiera. (Doctoral dissertation, University of Florida, 2009).
Primulaceae	Martins, L., Oberprieler, C. & Hellwig, F. A phylogenetic analysis of Primulaceae sl based on internal transcribed spacer (ITS) DNA sequence data. *Plant Syst. Evol.* **237**, 75-85 (2003).
Proteaceae	Barker, N.P., Weston, P.H., Rutschmann, F. & Sauquet, H. Molecular dating of the 'Gondwanan'plant family Proteaceae is only partially congruent with the timing of the break-up of Gondwana. *J. Biogeogr.* **34**, 2012-2027 (2007).
Ranunculaceae	Wang, W., Lu, A.-M., Ren, Y., Endress, M.E. & Chen, Z.-D. Phylogeny and classification of Ranunculales: evidence from four molecular loci and morphological data. *Perspect. Plant Ecol.* **11**, 81-110 (2009).
Rhamnaceae	Richardson, J.E., Fay, M.F., Cronk, Q.C., Bowman, D. & Chase, M.W. A phylogenetic analysis of Rhamnaceae using rbcL and trnL-F plastid DNA sequences. *Am. J. Bot.* **87**, 1309-1324 (2000).
Rubiaceae	Backlund, M. Phylogenetic Studies in the Gentianales–Approaches at Different Taxonomic Levels. (Doctoral dissertation, Uppsala University, 2005).
	Bremer, B. Phylogenetic studies within Rubiaceae and relationships to other families based on molecular data. *Opera Bot.*

Taxa	References
	Belg. **7**, 33-50 (1996).
Rutaceae	Chase, M.W., Morton, C.M. & Kallunki, J.A. Phylogenetic relationships of Rutaceae: a cladistic analysis of the subfamilies using evidence from RBC and ATP sequence variation. *Am. J. Bot.* **86**, 1191-1199 (1999).
	Groppo, M., Pirani, J.R., Salatino, M.L., Blanco, S.R. & Kallunki, J.A. Phylogeny of Rutaceae based on twononcoding regions from cpDNA. *Am. J. Bot.* **95**, 985-1005 (2008).
Scrophulariaceae	Olmstead, R.G., Wolfe, A.D., Young, N.D., Elisons, W.J. & Reeves, P.A. Disintegration of the Scrophulariaceae. *Am. J. Bot.* **88**, 348-361 (2001).
Solanaceae	Olmstead, R.G., Bohs, L., Migid, H.A., Santiago-Valentin, E., Garcia, V.F. & Collier, S.M. A molecular phylogeny of the Solanaceae. *Taxon* **57**, 1159-1181 (2008).
Verbenaceae	Wagstaff, S.J. & Olmstead, R.G. Phylogeny of Labiatae and Verbenaceae inferred from rbcL sequences. *Syst. Bot.* **22**, 165-179 (1997).
Xanthorrhoeaceae	Chase, M.W., De Bruijn, A.Y., Cox, A.V., Reeves, G., Rudall, P.J., Johnson, M.A. & Eguiarte, L.E. Phylogenetics of Asphodelaceae (Asparagales): an analysis of plastid rbcL and trnL-F DNA sequences. *Ann. Bot.* **86**, 935-951 (2000).
Zygophyllaceae	Sheahan, M.C. & Chase, M.W. Phylogenetic relationships within Zygophyllaceae based on DNA sequences of three plastid regions, with special emphasis on Zygophylloideae. *Syst. Bot.* **25**, 371-384 (2000).

Supplementary Table 4 Results of four phylogenetic logistic and linear regressions testing the global naturalization of alien species in relation to selfing ability using one average index per species (measured as self-compatibility and autofertility indices), native range size, annual/biennial vs. perennial life history, and the interaction between life history and selfing ability, using one index per species.. Global naturalization was measured as naturalization incidence outside the native range (expressed as being naturalized somewhere or not) and naturalization extent (natural log-transformed number of regions where the species is naturalized). Native range size was measured as the number of TDWG level-2 regions. Self-compatibility and autofertility indices were calculated using fruit set from our global breeding-system database. Sample sizes refer to the total number of species (one average index per species was used).

[a]Phylogenetic logistic regression, alpha=0.032 in the analysis with self-compatibility, and 0.025 in the analysis with autofertility.

[b]Phylogenetic linear model, lambda=0.200 in the analysis with self-compatibility and <0.0001 in the analysis with autofertility.

[c]Variables were centered and scaled. [d]Total explained variance by the variables in the model, and the relative importance of each variable calculated as the difference in deviance between the full model and a model without the variable of interest. R^2 could not be calculated for the phylogenetic logistic regressions.

Response variables	Naturalization incidence[a]				Naturalization extent[b]				
Explanatory variables	Estimates	SE	z	p	Estimates	SE	t	p	R^{2d}
Analysis with self-compatibility	*n* = 1115				*n* = 274				0.14
Intercept	-1.121	0.133	-8.41	<0.0001	2.007	0.215	9.32	<0.0001	-
Native range size (no. regions)[c]	0.826	0.090	9.21	<0.0001	0.119	0.100	1.18	0.237	6.18
Annual/biennial[c]	0.481	0.420	1.15	0.251	1.501	0.467	3.21	0.001	66.73
Self-compatibility index[c]	-0.002	0.076	-0.03	0.975	0.217	0.113	1.92	0.055	24.25
Annual/biennial[c] x Self-compatibility[c]	0.256	0.362	0.71	0.478	-0.356	0.446	-0.80	0.426	2.84
Analysis with autofertility	*n* = 828				*n* = 195				0.18
Intercept	-1.271	0.173	-7.35	<0.0001	2.369	0.130	18.21	<0.0001	-
Native range size (no. regions)[c]	0.846	0.107	7.93	<0.0001	0.015	0.121	0.12	0.903	0.07
Annual/biennial[c]	0.737	0.359	2.05	0.040	1.148	0.381	3.01	0.003	51.54
Autofertility [c]	0.107	0.086	1.24	0.215	0.431	0.133	3.23	0.001	48.18
Annual/biennial[c] x Autofertility[c]	0.336	0.249	1.5	0.177	-0.063	0.302	-0.21	0.834	0.21
